# Prevalence and factors associated with neck, shoulder and low back pains among medical students in a Malaysian Medical College

**DOI:** 10.1186/1756-0500-6-244

**Published:** 2013-07-01

**Authors:** Mustafa Ahmed Alshagga, Amal R Nimer, Looi Pui Yan, Ibrahim Abdel Aziz Ibrahim, Saeed S Al-Ghamdi, Sami Abdo Radman Al-Dubai

**Affiliations:** 1 Division of Biomedical Sciences, Faculty of Medicine, CUCMS, Cyberjaya, Selangor, 63000, Malaysia; 2 Newcastle University Medicine Malaysia, Kota Ilmu Educity@Iskandar, Nusajaya, Johor, 79200, Malaysia; 3 Faculty of Pharmacy, CUCMS, Cyberjaya 63000, Selangor, Malaysia; 4 Department of Pharmacology and Toxicology, Faculty of Medicine, Umm Al-Qura University, Makkah, Saudi Arabia; 5 Department of Community Medicine, International Medical University, Kuala Lumpur, Malaysia

**Keywords:** Musculoskeletal pain, Medical students, Malaysia

## Abstract

**Background:**

The main purpose of the study was to assess the prevalence, body distributions and factors associated with musculoskeletal pain (MSP) among medical students in a private Malaysian medical college.

**Method:**

This cross-sectional study was conducted among 232 medical students in a private medical college using an online questionnaire. The questionnaire was a modified Standardized Nordic Questionnaire focused on neck, shoulder and low back pain in the past week and the past year.

**Results:**

Two hundred and thirty two medical students responded to the questionnaire out of 642. Mean age was 20.7 ± 2.1 years. The majority were female (62.9%), Malay (80.6%) and in the preclinical years (72%). One hundred and six (45.7%) of all students had at least one site of MSP in the past week and 151 (65.1%) had at least one site of MSP in the past year. MSP in the past week was associated significantly with the academic year, (OR 2.0, 95% CI 1.15-3.67, P = 0.015), history of trauma (OR 2.6, 95% CI 1.2-5.3, P = 0.011), family history of MSP (OR 2.1, 95% CI 1.1-3.9, P = 0.023) and Body Mass Index (BMI) (P = 0.028). MSP in the past year was significantly associated with computer use (P = 0.027), daily hours of computer use (median ± IQR (5.0 ±3.0), history of trauma (OR 7.5, 95% CI 2.24-2.56, P < 0.01) and family history of MSP (OR 2.5, 95% CI 1.31-4.90, P = 0.006). On multivariate analysis, factors associated with MSP during the past week were a family history of MSP (p = 0.029) and BMI (p = 0.03). Factors associated with MSP during the past year were being in clinical years (p = 0.002, computer use (p = 0.038), and a history of trauma (p = 0.030).

**Conclusion:**

MSP among medical students was relatively high, thus, further clinical assessment is needed in depth study of ergonomics. The study results indicate that medical school authorities should take measures to prevent MSP due to factors related to medical school. Students should make aware of importance of weight reduction to reduce MSP.

## Background

The goals of a medical school are to produce competent, professional doctors and promote health care of society. But during the period of medical training, students are exposed to stress, study problems, long training hours in hospital wards and clinics [1,2]; in addition to the increasing use of computers in teaching and learning [3]. These events are considered modifiable musculoskeletal pain (MSP) risk factors that may increase the prevalence of MSP among medical students. MSP is a major cause of chronic pain, injury, illness, reduced educational attainment that may affect the quality of productivity, and absenteeism from university lessons [4] which will affect students’ future careers.

Derek Smith published several studies (2003–2005) investigating the prevalence, body distributions and risk factors of MSP among Asian undergraduate nurse and medical students. In these studies, the prevalence rates were different from country to country. Korean nurse students showed (73.3%) [5], Japanese nurse students (36.9%) [6] and Chinese medical student (67.6%) [7]. Smith’s studies among undergraduates also showed differences in body distribution, shoulder pain was predominate among nurse students and low back pain was the highest among medical students. Nevertheless, MSP studies which had been done among nurses at a workplace in Korea (93.6%) [8] and Japan (85.5%) [9] have shown a higher prevalence rate in comparison to nurse undergraduates in these countries. Debating occupational risk factors that would increase the prevalence in the workplace are out of the concern of this article. But it is important to mention Hanvold et al. [10] cohort study which reported MSP complaint at the first year of working life was three times higher than MSP complaint reported at baseline during student life.

The prevalence of upper limb symptoms reported among Malaysian office workers (34%) and its association to computer use, [11] while low back pain among Malaysian teachers (40%) was associated with physical activity [12]. A recent study [13] investigated the prevalence of MSP among Malaysian college students and its relationship to computer use. This study reported high prevalence (90%) among females and (76%) male students, but showed no link of MSP to computer use.

Therefore, it is important for medical schools to identify the possible modifiable MSP risk factors and plan early supportive and preventive measures for a better quality of life for future doctors. In so doing, we have to explore the magnitude of the problem in our school, which is a private funded medical college adopting the student-centered learning curriculum. This type of teaching is based on students preparing most of their learning material using the computer. Moreover, students of 3rd year and onwards are involved in hospital training which requires them to be engaged in physical activity. This study aimed to determine the prevalence of MPS among medical students in a Malaysian medical school.

## Methods

### Study design & participants

Using a universal sampling, this cross-sectional study was carried out among all medical students (n = 642) in a private medical college in Selangor, Malaysia during the period of March 2010 until October 2010.

### Study instruments

This study used an online self-administered questionnaire in English language adapted from the Standardized Nordic Questionnaire [14]. The questionnaire was modified, as described in Smith et al. [7], to facilitate comparison of our results with other medical students in the literature. The questionnaire was composed of three sections. Section A, included questions on socio-demographic data. Section B, included questions on the risk factors such as exercise and caffeine consumption, history of physical\trauma and family history of musculoskeletal disorders. Section C, included questions on the occurrence of pain in the neck, shoulders and low back during the past 7 days and the past 12 months. The questionnaire was pre-tested on 10 students before distributing to the subjects to ensure that the questionnaire was easily understood.

### Data collection

The web link of the questionnaire was distributed among the students, of all academic years, through their batch e-mails with the cover letter. The cover letter informed the participants of the purpose of the study, the assurance of anonymity and the entitlement of the respondents to complete or decline the survey questionnaire. Approval of the study was obtained from the faculty of Pharmacy Research Committee (Cyberjaya University College of Medical Sciences).

### Statistical analysis

Analysis was performed using Statistical Package of Social Sciences (SPSS) software version 17. Descriptive analysis was conducted to obtain the frequencies, mean, SD and median. Test of Normality was conducted for the continuous variables. The frequency and percent of the MSP in the past week and in the past year for each site was obtained (neck, shoulder and back). There were two dependent variables in this study; the first was ''MSP in the past week at least in one site” and the second was '' MSP in the past year at least in one site”. To assess the relationship between the dependent variables and the categorical independent variables, Chi square test was conducted to obtain the crude odds ratio and 95% CI. Simple logistic regression analysis was conducted for the variables with more than two categories. Mann–Whitney, a non-parametric test, was conducted to identify the difference between the dependent variables and the continuous independent variables. Multivariate binary logistic regression analysis was conducted for each of the dependent variables separately. Factors associated with the dependent variables in the bivariate analysis were included in the multivariate analysis.

## Results

Two hundred and thirty two students out of 642 participated in this study (response rate 36.13%). Mean (±SD) age was 20.6 (± 2.2) years and the majority there were females (72.9%). Socio-demographic data are shown in Table 1.

**Table 1:** Demographic characteristics of participants

**Variable**		**N (232)**		**%**
Gender	Male	68		37.1
	Female	146		62.9
Race	Malay	187		80.6
	Chinese	25		10.8
	Indian	20		8.6
Academic year	1	133		57.3
	2	34		14.7
	3	7		3.0
	4	40		17.2
	5	18		7.8
History of trauma in the neck, shoulder or low back	Yes	37		15.9
	No	195		84.1
Family history of MSD	Yes	88		37.9
	No	74		31.9
	Not sure	77		30.2
Exercise	Regularly	56		24.1
	Not regularly	153		65.9
	Not at all	23		10.0
Coffee	< 3 cups/week	147		63.4
	≥ 3cups/week	85		36.6
			**Mean (SD)**	
Body Mass Index			22.8 (4.4)	
Height			163.2 (10.3)	
Weight			61.5 (14.9)	
Hours of computer use/day			4.8 (2.8)	
Hours of study/day			3.5 (1.7)	

The prevalence of musculoskeletal pain (at least in one body site) was 45.7% in the past week and 65.1% in the previous year. The prevalence of low back pain was the highest in the past week and in the previous year (27.2%, 46.1% respectively), followed by neck pain (24.1%41.8% respectively) (Table 2).

**Table 2:** Prevalence of MSP during past week and past 12 months

**MSP Body site**		**Prevalence during past week**		**Prevalence during past 12 months**	
		**N**	**%**	**N**	**%**
	No	126	54.3	81	34.9
Neck pain	Yes	56	24.1	97	41.8
	No	176	75.9	135	58.2
Shoulder pain	Yes	20	8.6	53	22.8
	No	212	91.4	179	77.2
Low Back pain	Yes	63	27.2	107	46.1
	No	169	72.8	125	53.9
Overall (at least one site)		106	45.7	151	65.1

In the past week the prevalence of MSP was higher among the students in the clinical years compared to those in the pre-clinical years (OR = 2.0, 95% C.I 1.15-3.67, p = 0.015), among those who had history of physical trauma compared to those who had no trauma (OR = 2.6, 95% C.I 1.22-5.29, p = 0.011) and among those who reported significant family history of MSD in comparison to those who did not (OR = 2.6, 95% C.I 1.22-5.29, p = 0.011). The BMI was higher among students who had MSP (median = 21.4, IQR = 4.2) compared to those who had no MSP (median = 22.4, IQR = 5.7) (p = 0.028) (Table 3).

**Table 3:** Factors associated with MSP during past week among medical students using chi square test

		**MSP during past week**			
		**Yes (%)**	**No (%)**	**OR (95%CI)**	**P-value**
Gender					
	Male	41 (47.7)	45 (52.3)		
	Female	65 (44.5)	81 (55.5)	1.1 (0.66-1.92)	0.641
Race					
	Malay	85 (46.0)	101 (54.0)		
	Chinese	12 (48.0)	13 (52.0)		
	Indian	8 (40.0)	12 (60.0)		0.852
Academic year					
	Preclinical	68 (40.7)	99 (59.3)		
	clinical	38 (58.5)	27 (41.5)	2.0 (1.15-3.67)	0.015
History of trauma					
	Yes	24 (64.9)	13 (35.1)		
	No	82 (42.1)	113 (57.9)	2.6 (1.22-5.29)	0.011
Family history of MSD*					
	Yes	50 (56.8)	38 (43.2)	2.1 (1.11-3.97)	0.023**
	No	29 (39.2)	45 (60.8)	1.0 (0.53-2.00)	0.939
	Not sure	27 (38.6)	43 (61.4)	1.00	
Exercise	Regularly	22 (39.3)	34 (60.7)		
	Not regularly	74 (48.4)	79 (51.6)		
	Not at all	10 (43.5)	13 (56.5)		0.493
Coffee					
	< 3 cups/week	61 (41.5)	86 (58.5)		
	≥ 3cups/week	45 (52.9)	40 (47.1)		0.092
		Yes (Median, IQR)	No (Median, IQR)**		
Body Mass Index		106 (21.4 ±4.2)	126 (22.4, 5.7)		0.028
Hours of computer use/day		106 (4.0 ±3.0)	126 (5.0, 3.0)		0.130
Hours of study/day		106 (3.0 ±3.0)	126 (3.0, 2.0)		0.389

* Simple logistic regression.

** Nonparametric (Mann–Whitney test).

The prevalence of MSP in the past year was higher among those who were in clinical years compared to those in preclinical years (OR = 3.5, 95% CI 1.7-7.3, p = < 0.001), those with history of trauma in comparison to students with no history of trauma (OR = 7.6, 95% CI 2.24-2.56, p = < 0.001and students with family history of MSD compared to those without family history (OR = 2.5, 95% CI 1.31-4.90, p = 0.006). Students with MSP showed significantly longer daily hours of computer use (median = 5.0, IQR = 3.0) compared to those without MSP (median = 4.0, IQR =2.5), (p = 0.027) (Table 4).

**Table 4:** Factors associated with MSP during past 12 months among medical students using chi square test

		**MSP during past 12 months**			
**Variables**		**Yes (%)**	**No (%)**	**OR (95%CI)**	**P-value**
Gender					
	Male	59 (68.6)	27 (31.4)		
	Female	92 (63.0)	54 (37.0)	1.3 (0.7-2.3)	0.388
Race					
	Malay	124 (66.3)	63 (33.7)		
	Chinese	16 (64.0)	9 (36.0)		
	Indian	11 (55.0)	9 (45.0)		0.595
Academic year					
	Preclinical	97 (58.1)	70 (41.9)		
	clinical	54 (83.1)	11 (16.9)	3.5 (1.7-7.3)	< 0.001*
History of trauma					
	Yes	34 (91.9)	3 (8.1)	7.6 (2.24-2.56)	< 0.001^
	No	117 (60.0)	78 (40.0)		
Family history of MSD*					
	Yes	65 (73.9)	23 (26.1)	2.5 (1.31-4.90)	0.006
	No	39 (52.7)	35 (47.3)	1.00	
	Not sure	47 (67.1)	23 (32.9)	1.8 (0.93-3.60)	0.079
Exercise*	Regularly	33 (58.9)	23 (41.1)	1.9 (0.69-4.97)	0.213
	Not regularly	108 (70.6)	45 (29.4)	3.1 (1.27-7.63)	0.013
	Not at all	10 (43.5)	13 (56.5)	1.00	
Coffee					
	< 3 cups/week	89 (60.5)	58 (39.5)	1.8 (0.98-3.14)	0.056
	≥ 3cups/week	62 (72.9)	23 (27.1)		
		Yes (Median, IQR)	No (Median, IQR)**		
Body Mass Index		(22.3, 5.2)	(21.3, 4.5)		0.052
Hours of computer use/day		(5.0, 3.0)	(4.0, 2.5)		0.027
Hours of study/day		(3.0 ±2.0)	(3.0, 2.0)		0.741

* Simple logistic regression.

** Nonparametric (Mann–Whitney test).

On multiple logistic regression analysis, factors associated with MSP during the past week were family history of MSD (OR = 1.8, 95% CI 1.0-3.2, p = 0.029) and increasing BMI (OR = 1.1, 95% CI 1.0-1.1, p = 0.03). The total model was significant (p < 0.001) and accounted for 10% of the variance (Table 5). Factors associated with MSP during the past year were being in the clinical years (OR = 3.5, 95% C.I 1.5-7.0, p = 0.002), daily hours of computer use (OR = 1.1, 95% C.I 1.1-1.2, p = 0.038) and history of physical trauma (OR = 6.5, 95%CI 2.0-22.7, p = 0.030). The total model was significant (p < 0.001) and accounted for 19% of the variance (Table 6).

**Table 5:** Multiple logistic regression of factors associated with MSP during the past week

**Variable**		**B**	***p *value**	**OR**	**95% CI**
Family history of MSD	Yes	0.617	.0291	1.8	1.0-3.2
	No			1.0	
History of Physical Trauma	yes	0.745	0.544	2.1	1.0-4.5
	No			1.0	
Body Mass Index (BMI)		0.070	0.030	1.1	1.0-1.1

B = Regression estimate; OR = the odds ratio; 95% CI = 95% Confidence Interval; ref = reference category.

**Table 6:** Multiple logistic regression of factors associated with MSP during the past year

**Variable**		**B**	***p *value**	**OR**	**95% CI**
Family history of MSD	Yes	0.432	0.172	1.5	0.9-2.8
	No			1.0	
History of Physical Trauma	yes	1.884	0.030	6.5	2.0-22.7
	No			1.0	
Academic year	Clinical	1.184	0.002	3.2	1.5-6.8
	Preclinical			1.0	
Computer use		0.112	0.038	1.1	1.0-1.2

B = Regression estimate; OR = the odds ratio; 95% CI = 95% Confidence Interval; ref = reference category.

## Discussion

Almost half of the undergraduates at this university reported having MSP in at least one body site. Reporting musculoskeletal pain during the past week 1-year apart however, does not necessarily mean that pain has been continuous. In our study, among the subjects having MSP, some may have had continuous pain while others may have had only consecutive pain episodes. Our current result is similar to a previous investigation done for undergraduate medical students [15]. In this study, medical students reported MSP most commonly at their lower back. The 12 months period prevalence (46.1%) was lower than that reported by Japanese orthopedic surgeon (50%) [16], but higher than that reported for Chinese Medical students (40.1%) [7].

Statistical analysis of our data provides some interesting findings. Basic investigations with Pearson’s Chi-Square test and Fisher’s exact test showed that most demographic items did not differ significantly between those students reporting MSP and those who did not. This is contrary to some previous studies among university students. An Australian study of nursing students, for example, revealed statistically significant difference in MSP prevalence rate between males and females, with males having the highest rates [17]. Similarly, research conducted upon American college students found that females incurred an elevated risk for MSP of the upper body [18].

Clinical practice was shown to be associated with MSP reporting among our students who may suggest the complicity of clinical training in hospitals. In this study, the participants in clinical years were recruited from various hospital postings such as Medicine, Surgery, Obstetrics and Pediatrics therefore, our instrument is limited in terms of measuring the physical activity related to each posting as specific risk factors were not sought. This study has showed that students in clinical years were twice as likely to have MSP during the past week (p = 0.015). Probably because students in clinical years spend more time in standing position during bedside teaching, clerking or attending surgical operation.

Participation in regular exercise showed no relationship with MSP, which is contrary to a previous study of American university students, where participation in athletics offers a protective effect against MSD of the upper body [18]. It is possible that this lack of association may have related to our broad categorization of the term ‘exercise’ rather than dividing it into separate levels such as walking, jogging, running and so on. As such, future longitudinal studies of MSP should be conducted among Malaysian students.

In the present study, we observed that an increasing duration of computer use was associated with MSP during the past week. Rajagopal et al. [13] reported high prevalence of MSP among Malaysian college students, but showed no association of MSP and computer use. Rajagopal study [13] had been done on a smaller sample size as mentioned in his study limitation. However, our results were in agreement with findings of other literature reviews [19,20].

Being in clinical years, history of trauma, positive family history, being overweight and long computing period was associated with higher prevalence of symptoms, normal BMI and adjusted hours of computing appeared to be protective. Jones et al. [21] set out to identify factors relating to persons who do not report musculoskeletal pain in the general adult population and found that good sleep quality, normal illness behavior, low psychological distress, and an absence of recent adverse life events characterized those without reported pain. These findings indicate that there are a variety of risk factors in this condition, as suggested by others [15,22]. The finding that greater exposure to computing is associated with higher symptom prevalence is consistent with prior work [19].

Suboptimal health behavior may also be an important consideration for medical students, as the previous researcher had described, young Australian doctors tended to give their health care a low priority [23]. This behavior might be the direct cause of MSP being more prevalent among clinical year’s students in our study. The occupational stressor of medical work and work posture of clinical year’s students might add another effect upon the MSP prevalence. It is conceivable therefore, that increased health promoting behavior among medical students may offer a protective effect against MSP at various body sites.

Coffee contains caffeine which helps in combating fatigue and drowsiness and alleviating pain. McPartland and Mitchell [24] reported high consumption of caffeine by patients with low back pain and discussed the importance of reducing coffee intake among patients with chronic low back pain, as caffeine increases urinary calcium and could have a detrimental effect on bones on long term.

An important and potentially preventable [25] public health problem may be emerging on college campuses. If the observed high prevalence of upper extremity disorders is confirmed, research on interventions for these disorders in students should receive high priority. Lo-ngitudinal studies are needed to delineate the natural history of these disorders, and studies involving younger students may be worthwhile to identify opportunities for intervention.

The present study indicates that there is a considerable subgroup of adolescents, which repeatedly reports comorbid musculoskeletal pain in several body locations, and that both psychosocial factors and factors related to lifestyle may explain the propensity for persistent multiple pains. The trajectories of pain development in adolescence and early adulthood, their risk factors and their determinants deserve further research.

### Limitations of the study

The results from this study are limited by the low response rate which, is probably due to the use of an online survey as well as the instrument being self-reported that can be affected by the subjective status of the students. The student centered curriculum in this study could be associated with some factors that increase risk of MSP, as it requires significant computer use in preclinical and clinical attachments.

Moreover the instrument which was used is limited to measure the ergonomic risk factors in detail that medical students could encounter during clinical postings. For these reasons, the results from this study cannot be generalized to all medical students. As stated in our introduction, the study was conducted to explore the magnitude of MSP among medical students rather than to study the detailed risk factors. Nevertheless the results are alarming, and careful attention should be given by medical schools to increase students’ awareness of MSP, weight reduction and provide some adjustment for computer lab in school as an appropriate work place measures. Moreover, medical school should evaluate the physical health of the students who are doing hazardous physical activity during clinical attachment, particularly the students with history of physical trauma or positive family history of musculoskeletal disorders.

## Conclusion

MSP among medical students was relatively high, thus, further clinical assessment is needed in depth study of ergonomics. The study results indicate that medical school authorities should take measures to prevent MSP due to factors related to medical school. Students should make aware of importance of weight reduction to reduce MSP.

## Competing interests

The authors declare that they have no competing interests.

## Authors’ contributions

MAA, LPY, SAA conceive the study. All the authors participate to design the study. LPY collected key in the data. ARN, IAI, SSA, LPY designed the online questionnaire. MAA, SAA did the statistical analysis. MAA, ARN, IAI, SAA wrote the manuscript. All the authors read and approved the manuscript.
